# Supplementary material for: Analysis of child development facts and myths using text mining techniques and classification models
Source: Heliyon. 2024 Aug 23;10(17):e36652. doi: 10.1016/j.heliyon.2024.e36652 (PMC11388728; doi:10.1016/j.heliyon.2024.e36652)
Supplement: Multimedia component 1 [file mmc1.docx]

We also experimented by increasing the number of classification labels. We categorized myths and facts into four categories: (i) Strong fact: Statements have a strong argument, (ii) Weak fact: Statements have a weak argument, (iii)Weak myth: Statements have a weak argument, (iv) Strong myth: Statements have a strong argument.

 According to the results shown in Table 4, TF-IDF was found to be more effective, with an accuracy of 69%, compared to BoW's accuracy of 66% for SVM. On the contrary, the second highest accuracy rate was 66% for LR, while using both TF-IDF and BoW. DT has the lowest accuracy (57%) using TF-IDF and KNN has the lowest accuracy (48%) using BoW. This suggests that the use of TF-IDF can enhance the performance of the SVM for the data. In the data, Strong Facts have the highest number of statements. As a result, the classification models have the highest percentages of precision, recall and F1-score for Strong Facts. SVM achieved the highest precision (79%) using BoW feature extraction and the highest F1-score (80%) for TF-IDF feature extraction. On the other hand, LR achieved the highest recall (94%) for TF-IDF feature extraction. KNN has the lowest precision (29%) for Strong Myths, the lowest recall (12%), and the lowest f1-score (20%) for Weak Facts using BoW. Support is 142 for Strong Facts, 52 for Strong Myths, 50 for Weak Facts and 35 for Weak Myths.

    We examined the result of classification by confusion matrix as illustrated in Fig. 5 and 6. In Figure 5, the confusion matrix for classification accuracy using TF-IDF is shown, while in Figure 6, the confusion matrix for classification accuracy using Bow is shown.

**Table 4**

Accuracy matrix for 4 labels

| Classifications | Labels | TF-IDF | | | | |  | BoW | | | | |  | |
| --- | --- | --- | --- | --- | --- | --- | --- | --- | --- | --- | --- | --- | --- | --- |
|  |  | **Precision** | **Recall** | **F1-score** | **Support** | **Accuracy** |  | **Precision** | **Recall** | **F1-score** | **Support** | **Accuracy** | |  |
| NB | Strong facts | 0.66 | 0.87 | 0.75 | 142 | 0.63 |  | 0.65 | **0.87** | 0.74 | 142 | 0.61 | |  |
|  | Strong myths | 0.52 | 0.27 | 0.35 | 52 |  |  | 0.55 | 0.31 | 0.4 | 52 |  |  |  |
|  | Weak facts | 0.56 | 0.56 | 0.56 | 50 |  |  | 0.52 | 0.5 | 0.51 | 50 |  |  |  |
|  | Weak myths | 0.67 | 0.29 | 0.4 | 35 |  |  | 0.58 | 0.2 | 0.3 | 35 |  |  |  |
|  | Macro avg | 0.6 | 0.5 | 0.52 | 279 |  |  | 0.58 | 0.47 | 0.49 | 279 |  |  |  |
|  | Weighted avg | 0.62 | 0.63 | 0.6 | 279 |  |  | 0.6 | 0.61 | 0.58 | 279 |  |  |  |
| SVM | Strong facts | **0.72** | 0.9 | 0.8 | 142 | **0.69** |  | **0.79** | 0.78 | **0.79** | 142 | **0.66** | |  |
|  | Strong myths | 0.55 | 0.52 | 0.53 | 52 |  |  | 0.52 | 0.58 | 0.55 | 52 |  |  |  |
|  | Weak facts | 0.74 | 0.52 | 0.61 | 50 |  |  | 0.67 | 0.58 | 0.62 | 50 |  |  |  |
|  | Weak myths | 0.69 | 0.31 | 0.43 | 35 |  |  | 0.37 | 0.4 | 0.38 | 35 |  |  |  |
|  | Macro avg | 0.67 | 0.56 | 0.59 | 279 |  |  | 0.59 | 0.58 | 0.58 | 279 |  |  |  |
|  | Weighted avg | 0.69 | 0.69 | 0.67 | 279 |  |  | 0.67 | 0.66 | 0.66 | 279 |  |  |  |
| LR | Strong facts | 0.67 | **0.94** | 0.78 | 142 | **0.66** |  | 0.76 | 0.82 | **0.79** | 142 | **0.66** | |  |
|  | Strong myths | 0.57 | 0.44 | 0.5 | 52 |  |  | 0.48 | 0.5 | 0.49 | 52 |  |  |  |
|  | Weak facts | 0.75 | 0.42 | 0.54 | 50 |  |  | 0.65 | 0.56 | 0.6 | 50 |  |  |  |
|  | Weak myths | 0.67 | **0.23** | 0.34 | 35 |  |  | 0.48 | 0.4 | 0.44 | 35 |  |  |  |
|  | Macro avg | 0.67 | 0.51 | 0.54 | 279 |  |  | 0.59 | 0.57 | 0.58 | 279 |  |  |  |
|  | Weighted avg | 0.67 | 0.66 | 0.63 | 279 |  |  | 0.65 | 0.66 | 0.65 | 279 |  |  |  |
| DT | Strong facts | 0.7 | 0.68 | 0.69 | 142 | 0.57 |  | 0.71 | 0.72 | 0.72 | 142 | 0.59 | |  |
|  | Strong myths | 0.46 | 0.52 | 0.49 | 52 |  |  | 0.43 | 0.56 | 0.48 | 52 |  |  |  |
|  | Weak facts | 0.54 | 0.44 | 0.48 | 50 |  |  | 0.61 | 0.5 | 0.55 | 50 |  |  |  |
|  | Weak myths | 0.32 | 0.37 | 0.34 | 35 |  |  | 0.3 | 0.23 | 0.26 | 35 |  |  |  |
|  | Macro avg | 0.5 | 0.5 | 0.5 | 279 |  |  | 0.51 | 0.5 | 0.5 | 279 |  |  |  |
|  | Weighted avg | 0.58 | 0.57 | 0.57 | 279 |  |  | 0.59 | 0.59 | 0.59 | 279 |  |  |  |
| RF | Strong facts | 0.68 | 0.87 | 0.76 | 142 | 0.64 |  | 0.71 | 0.84 | 0.77 | 142 | 0.63 | |  |
|  | Strong myths | 0.47 | 0.48 | 0.48 | 52 |  |  | 0.44 | 0.56 | 0.49 | 52 |  |  |  |
|  | Weak facts | 0.74 | 0.46 | 0.57 | 50 |  |  | 0.79 | 0.44 | 0.56 | 50 |  |  |  |
|  | Weak myths | 0.5 | 0.2 | 0.29 | 35 |  |  | 0.41 | 0.2 | 0.27 | 35 |  |  |  |
|  | Macro avg | 0.6 | 0.5 | 0.52 | 279 |  |  | 0.59 | 0.51 | 0.52 | 279 |  |  |  |
|  | Weighted avg | 0.63 | 0.64 | 0.61 | 279 |  |  | 0.63 | 0.63 | 0.62 | 279 |  |  |  |
| KNN | Strong facts | 0.67 | 0.81 | 0.73 | 142 | 0.61 |  | 0.69 | 0.63 | 0.66 | 142 | 0.48 | |  |
|  | Strong myths | 0.45 | 0.46 | 0.46 | 52 |  |  | 0.29 | 0.71 | 0.41 | 52 |  |  |  |
|  | Weak facts | 0.72 | 0.42 | 0.53 | 50 |  |  | 0.67 | 0.12 | 0.2 | 50 |  |  |  |
|  | Weak myths | **0.42** | 0.31 | 0.36 | 35 |  |  | **0.09** | **0.03** | **0.04** | 35 |  |  |  |
|  | Macro avg | 0.57 | 0.5 | 0.52 | 279 |  |  | 0.43 | 0.37 | 0.33 | 279 |  |  |  |
|  | Weighted avg | 0.61 | 0.61 | 0.6 | 279 |  |  | 0.53 | 0.48 | 0.45 | 279 |  |  |  |

As described in Table 5, the experiment showed that the SVM algorithm achieved the highest accuracy rate (65%) using TF-IDF feature extraction, followed by the LR algorithm, which achieved 63% using both TF-IDF and BoW. These results show that SVM and LR performed well on our dataset. For TF-IDF, DT has the lowest accuracy (54%), while KNN has the lowest accuracy (44%) for BoW feature extraction.

**Table 5**

Cross-fold validation for 4 labels

| Feature Extraction | Classifications | Cross Fold Validation | | |
| --- | --- | --- | --- | --- |
|  |  | **K fold** | | **Leave one out** |
|  |  | **5-Fold** | **10-Fold** |  |
| TF-IDF | **NB** | 0.51 | 0.52 | 0.55 |
|  | **SVM** | 0.55 | 0.56 | **0.65** |
|  | **LR** | 0.54 | 0.55 | **0.63** |
|  | **DT** | 0.45 | 0.46 | 0.54 |
|  | **RF** | 0.53 | 0.53 | 0.62 |
|  | **KNN** | 0.49 | 0.48 | 0.6 |
| BoW | **NB** | 0.59 | 0.6 | 0.61 |
|  | **SVM** | 0.6 | 0.61 | 0.62 |
|  | **LR** | 0.63 | 0.64 | **0.63** |
|  | **DT** | 0.53 | 0.54 | 0.54 |
|  | **RF** | 0.62 | 0.62 | 0.62 |
|  | **KNN** | 0.43 | 0.43 | 0.44 |


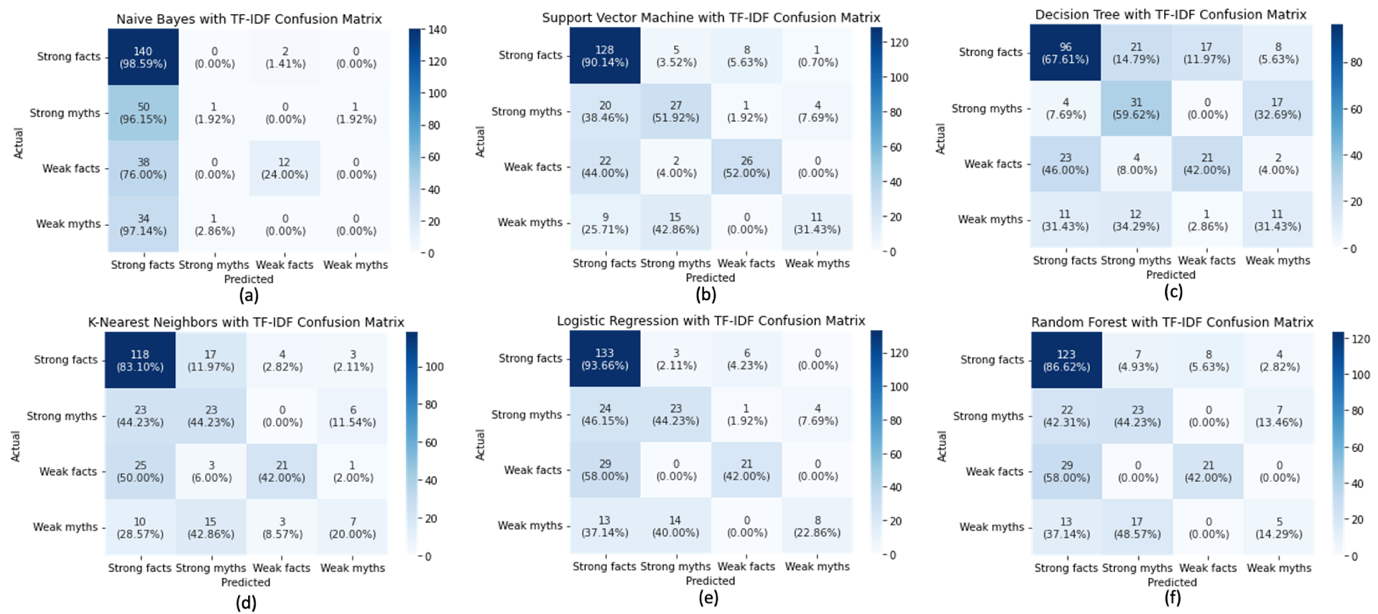


**Fig. 5.** Confusion matrix for TF-IDF for 4 labels.


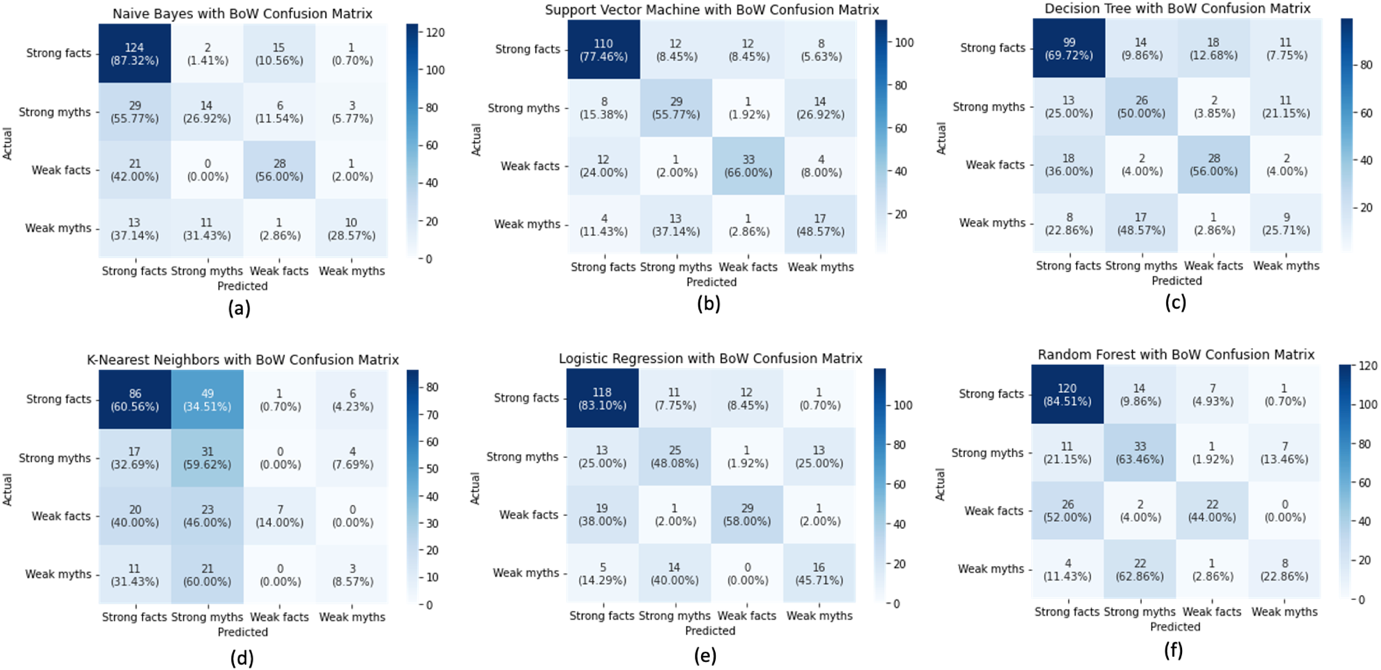


**Fig. 6.** Confusion matrix for BoW for 4 labels.
